# Source analysis of heavy metal pollution in agricultural soil irrigated with sewage in Wuqing, Tianjin

**DOI:** 10.1038/s41598-021-96367-8

**Published:** 2021-09-08

**Authors:** Jingran Wang, Danyang Yu, Yanhong Wang, Xueli Du, Guochen Li, Bo Li, Yujie Zhao, Yinghui Wei, Shuang Xu

**Affiliations:** 1 Institute of Applied Ecology, Chinese Academy of Sciences, Shenyang, 110016 China; 2 Key Laboratory of Pollution Ecology and Environmental Engineering, Institute of Applied Ecology, Chinese Academy of Sciences, Shenyang, 110016 China; 3 Liaoning Engineering Technology Research Center of Agricultural Products Quality and Environment Safety Control, Institute of Applied Ecology, Chinese Academy of Sciences, Shenyang, 110016 China; 4 Key Laboratory for Environmental Factors Control of Agro-Product Quality Safety, Ministry of Agriculture and Rural Affairs, Tianjin, 300191 China; 5 School of Chemical Engineering, Shenyang University of Chemical Technology, Shenyang, 110142 China

**Keywords:** Environmental sciences, Natural hazards

## Abstract

In this study, the contents of heavy metals and Cd and Pb isotope ratios of agricultural soil and potential source samples collected from farmland receiving sewage irrigation in Wuqing District, Tianjin, China were determined. Multiple methods were used for source analysis, including positive matrix factorization (PMF), correlation analysis, principal component analysis (PCA), and the Cd and Pb isotope ratio method. The results showed that agricultural soil was slightly contaminated by heavy metals in the research area, with relatively higher Cd and Pb accumulation levels compared to those of other heavy metals. Four types of pollution sources, including the soil parent material sources, industrial emission sources, agricultural practice sources, and mixed sources of sewage irrigation and transportation were apportioned and quantified by PMF, combined with the results of PCA and correlation analysis. The contribution rates quantified by the Cd and Pb isotope ratio method were similar, suggesting that no single source dominates Pb and Cd pollution. The contribution rates of Pb analyzed by the isotope ratio method were almost identical to those of the PMF model, indicating the rationality of the PMF result. Our results suggested that correlation analysis and PCA should be utilized to provide information for obtaining reasonable results and defining source categories for PMF, whereas the isotope ratio method should be applied to verify the accuracy of source contributions analyzed by PMF.

## Introduction

With the rapid development of urbanization and industrialization in the past few decades, agricultural soil has been contaminated by different pollutants, including heavy metals^1^. Heavy metals, which are defined as “naturally occurring metals having an atomic number greater than 20 and an elemental density greater than 5 g·cm^−3^”^2^, have always been a topic of considerable research interest due to their limited biodegradability and adverse health effects^3^. Different types of human sources, including industrial production, motor vehicle exhaust, sewage irrigation of farmland, ore mining and smelting, and fertilizer application^4–7^, exhibit significant impacts on heavy metal contents in the soil environment. Exploring the sources of heavy metals in soil is the key to controlling and preventing soil pollution.

Many methods, including the isotope ratio method, multivariate statistical analysis method, receptor model method, and chemical mass balance method^8–11^, have been utilized to analyze the sources of heavy metal pollution in recent decades. These methods have advantages and disadvantages in practical applications^12–14^. The stable isotope composition of a heavy metal is a natural attribute and unique marker that can be used to identify the sources of heavy metal pollution efficiently and accurately^15,16^. For example, Pb isotopes have been widely used to analyze the sources of Pb in air, biota, ore, sediment, soil, and water due to their stable physical and chemical properties^16–20^. Other stable isotopes, such as those of Cd, Cu, Fe, Hg, and Zn, were also used to identify pollution sources^20–23^. With the rapid development of Cd isotope determination technology in recent years, isotopic composition has been applied successfully to characterize the isotope fractionation produced by industrial activities and other processes and to provide information for tracing the sources of Cd in the environment^22–24^. Multivariate statistical analysis methods include correlation analysis and principal component analysis (PCA)^25–27^. Correlation analysis is used to explore the interaction between different elements^28–30^, considering that high correlation coefficients between elements indicate that the corresponding elements shared similar pollution sources^31^. PCA can categorize pollutants by extracting a few principal components (PCs) from among many observations and has been widely used to characterize heavy metal pollution^32–34^. Positive matrix factorization (PMF) is one of the most widely used receptor models recommended by the Environmental Protection Agency of the United States (USEPA). Previously, it was applied to analyze the sources of air pollutants^35^ and has been gradually used to analyze the sources of heavy metals in agricultural soil in recent years^36,37^.

Wuqing District in Tianjin, China, is an intensive agricultural area that has been irrigated with sewage for over 50 years. In previous studies conducted in this area, anthropogenic activity including industrial activities (i.e., electroplating, metallurgy, and chemical industry), application of fertilizers (i.e., chemical fertilizers and livestock manure), sewage irrigation, and transportation, contributed to the heavy metal pollution of the agricultural soil^38,39^. Appropriately applying different source analysis methods in different polluted areas, especially for the Wuqing sewage irrigation district, is the key to obtaining comprehensive information on the characteristics of heavy metal sources. The main objectives of this study were (1) to identify sources of heavy metals and quantify their contributions to the level of each heavy metal by using the PMF model combined with correlation analysis, PCA, and isotope ratio method and (2) to establish an appropriate pattern for applying source analysis methods in the Wuqing sewage irrigation district. This study will provide the basis for further analysis of the sources of pollutants in the agricultural soil of Wuqing and other areas exhibiting similar pollution patterns.

## Methods

### Research area

The research area is Wuqing District, which is located in northwestern in Tianjin, the middle and lower reaches of the Haihe River basin (39.48° N–39.59° N, 116.92° E–117.09° E). Its climate represents a semihumid continental monsoon with an average temperature of 11.63 °C and four distinct seasons. The soil types of research area are Eutric Cambisols and Gleyic Cambisols with a deep soil horizon that is cultivated by wheat, corn, and vegetables. These cultivated lands are distributed intensively and contiguously and are adjacent to roads, industries, and crisscrossed rivers. Peng et al. conducted a field study in Wuqing District and analyzed the influence of different sources on the level of heavy metals, including long-term sewage irrigation, industrial emission, agricultural inputs and soil parent material^39^. The agricultural soil in this research area was contaminated by multiple pollution sources.

## Soil sampling and chemical analysis

### Soil sampling

**Figure 1:**
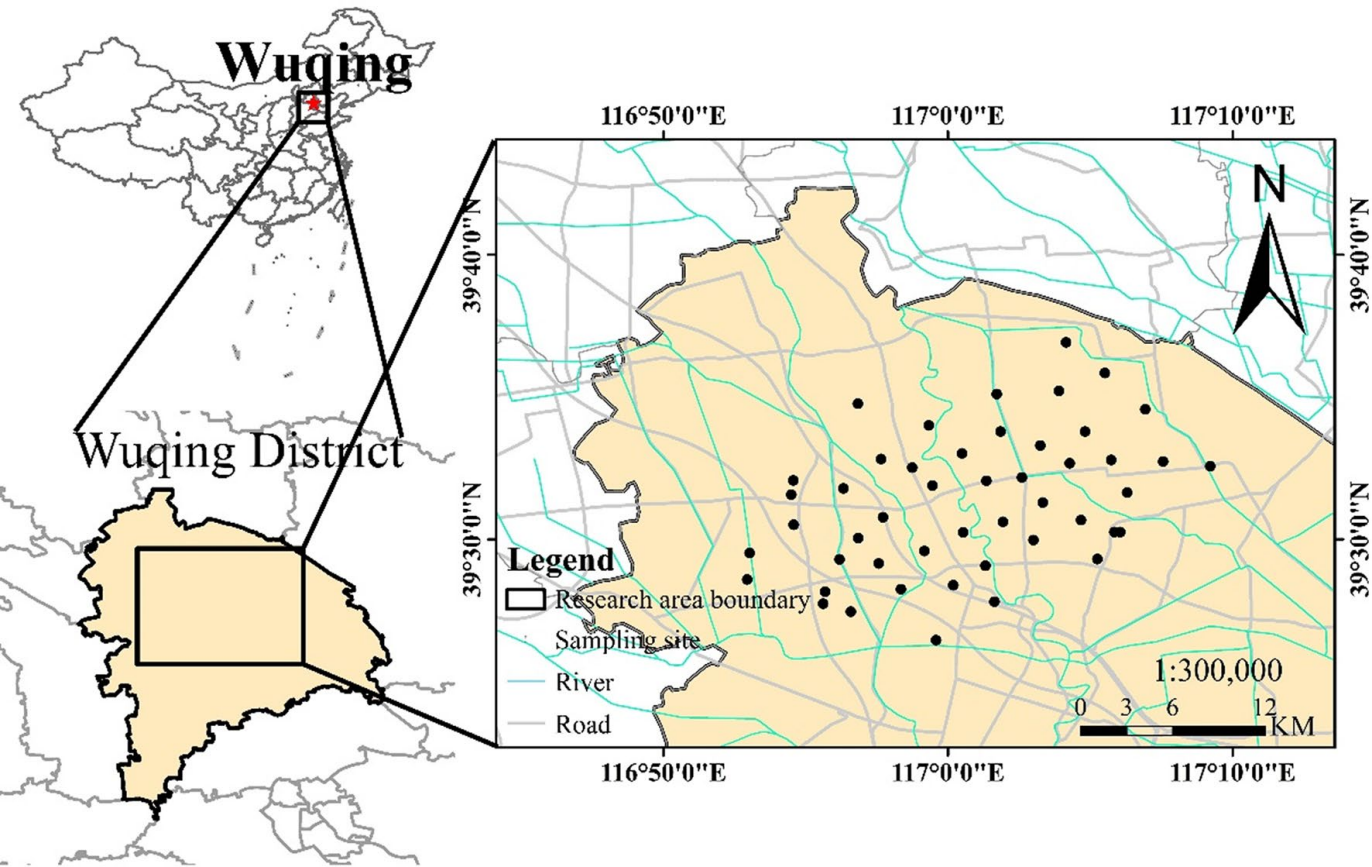
Sampling points in the research area of Wuqing, Tianjin (created by Arcgis 10.2, https://desktop.arcgis.com/en/).

Figure 1 shows the sampling locations established in the sewage irrigation area of Wuqing, Tianjin. Forty-eight topsoil samples (0–20 cm) were collected from each sampling site. Five samples of each potential source, including parent material soil (40–60 cm), factory soil, agricultural fertilizers, automobile exhaust dust, and irrigation sewage in the research area, were collected simultaneously. The agricultural fertilizer we collected is the most commonly used fertilizer in Wuqing District. The automobile exhaust dust was swept from exhaust pipe from heavy vehicles and private cars.

### Chemical analysis

The contents of thirteen elements and Cd and Pb isotope ratios were measured by inductively coupled plasma mass spectrometry (ICP-MS) and multicollector inductively coupled plasma mass spectrometry (MC-ICP-MS), respectively. Prior to determinations, soil samples were air-dried at room temperature (approximately 25 °C) and homogenized. A quarter of the soil sample was sieved through a 2 mm nylon sieve for the determination of pH using the procedure described in the Chinese Determination Standard of Soil pH (HJ 962-2018). Another quarter of the soil sample was ground in an agate mortar and then sieved through a 0.15 mm nylon sieve for the determination of element contents and Cd and Pb isotope ratios. Dust and fertilizer samples were subjected to the same pretreatment procedure used with soil samples. According to the digestion procedure of the National Soil Environmental Quality Standard in China (CNS, GB 15618-2018), each sample was digested with 10 ml of mixed acids (HNO_3_:HClO_4_ = 4:1) and 5 ml of hydrofluoric acid, after which the digested solutions were heated to approximately dryness on a heating plate. The digested solution was cooled and diluted to 25 ml volume with high-pure water. The contents of Ca, Cd, Cr, Cu, Fe, Mg, Mn, Ni, P, Pb, Ti, V, and Zn were determined by ICP-MS (NexION 300X). Quality assurance and control was performed by using blind triplicates and soil standard reference materials (GSS-14 and GSS-16, China National Center for Standard Material). The relative standard deviations (RSDs) of triplicate samples were between 4 and 6%. The relative errors (REs) between the measured and certified values of the standard reference materials were below 10%.

The determination of the Pb isotope ratio was done by following the method of Yoo et al.^40^ Briefly, the digestion solution containing at least 200 ng of Pb should be measured to meet the requirement of Pb isotope determination. The anion exchange resins were first equilibrated with 1 ml hydrochloric acid (1 mol/L). Then, the approximately dried digestion solutions for each sample were dissolved in 1 ml hydrochloric acid (1 mol/L), after which the anion exchange resins were rinsed with the dissolved digestion solutions. Finally, we eluted the solutions with 1 ml high-purity water (Milli-Q), heated these eluents on a heating plate to approximately dryness, and diluted them to 2 ml with 2% nitric acid. MC-ICP-MS (Nu Plasma, China Institute of Metrology) was utilized to determine the Pb isotope ratios, including ^206^Pb, ^207^Pb, and ^208^Pb. Quality control was performed with the Pb isotope SRM 981 (NIST981) obtained from the American National Standards Institute.

The Cd isotopes, including ^111^Cd, ^112^Cd, and ^114^Cd, were also determined by MC-ICP-MS. The procedure for determination of Cd isotopes was almost identical to that used for Pb isotopes. Each digestion solution should be measured to contain no less than 200 ng of Cd. Hydrochloric acid (2 mol/L) was used in Cd isotope determination, and the Cd isotope standard reference material (GBW04622) obtained from the Center of National Standard Reference Material was tested for quality control.

### Data analysis

Basic statistical data analyses and plots were conducted with Excel 2010 and Origin 2017, heavy metal pollution assessment was performed by the geoaccumulation index (Igeo), source analysis was preceded with correlation analysis and PCA from SPSS 22, PMF 5.0, and IsoSource software of the EPA.

### Igeo

Igeo allows characterization of the influence of exogenous inputs on the level of heavy metal pollution^41^, and it is calculated by Eq. (1):

1 $$\text{Igeo} = {\text{log}}_{2} \left(\frac{{\text{C}}_{\text{n}}}{{\text{1.5B}}_{\text{n}}} \right)$$

where Cn is the element content of the soil sample and Bn is the background level of the corresponding element in Tianjin soil^42^. The classification of Igeo is shown in Table 1.

**Table 1:** Classification with Igeo.

Igeo value	Soil quality
≤ 0	Unpolluted
0–1	From unpolluted to moderately polluted
1–2	Moderately polluted
2–3	From moderately to strongly polluted
3–4	Strongly polluted
4–5	From strongly to extremely polluted
5–6	Extremely polluted

### PMF model

The PMF model calculates the uncertainty of each chemical component in samples by weight and then employs the least squares method to apportion the source and quantify the contributions of pollutants. The contributions of sources to each component are constrained to positive values, and error estimates are applied for individual weightings of data points, relying on more physically significant assumptions. According to the EPA PMF 5.0 User Guide, the contribution of each source is calculated by Eq. (2):

2 $${\text{x}}_{\text{ij}}\text{ = }{\sum }_{\text{k=1}}^{\text{p}}{{\text{g}}}_{\text{ik}}{{\text{f}}}_{\text{ kj}}\text{ + }{\text{e}}_{\text{ij}}$$

where x_ij_ is the content of element j in sample i, g_ik_ is the contribution of source k to sample i, f_kj_ is the mass fraction of element j in source k, and e_ij_ is the residual matrix, which is excluded from the model.

To obtain the appropriate factor profiles and factor contributions, the objective function Q will be minimized. Q is calculated by Eq. (3):

3 $${{\text{Q}} = \sum_{\text{i=1}}^{\text{n}}\sum_{\text{j=1}}^{\text{m}}}{\left(\frac{{{\text{x}}_{\text{ij}}{-\sum}}_{\text{k=1}}^{\text{p}}{{\text{g}}}_{\text{ik}}{{\text{f}}}_{\text{kj}}}{{\text{u}}_{\text{ij}}}\right)}^{2}$$

where u_ij_ is the uncertainty of element j in sample i and is calculated by Eqs. (4) and (5):

4 $$\text{Unc  } =\frac{5}{{6}}\,{\times}\,\text{MDL (c } \,\leq\, {\text MDL)}$$

5 $$\text{Unc} = \sqrt{{\sigma \times \text{(c)}}^{2}{+}{\text{MDL}}^{2}}\text{ (c }{>}\text{ MDL)}$$

### EPA-IsoSource

The EPA-IsoSource model is based on the binary and ternary mixing model, using the principle of conservation of mass and repeated calculation rules to produce a combination of multiple source ratios. According to previous studies^43,44^, the calculation is performed with Eq. (6):

6 $$\text{Q  } = \frac{\text{(}\frac{100}{{\text{i}}}\text{)+(s-1)}}{\text{s-1}}\,{ = }\,\frac{\left[\text{(}\frac{100}{{\text{i}}}\text{)+(s-1)}\right]\text{!}}{\text{(}\frac{100}{{\text{i}}}\text{)!(s-1)!}}$$

where i is the increment (%) and s is the number of sources.

## Results and discussion

### Descriptive statistics and assessment of heavy metals

Descriptive statistics of heavy metal contents are shown in Table 2. The screening values of Cd, Cr, Cu, Ni, Pb, and Zn determined in CNS with pH values ranging from 6.5 to 7.5 were selected to assess the pollution levels of these heavy metals, since the average pH of the soil samples was 7.2. Among all soil samples, the Cd contents of eight samples exceeded the corresponding threshold. Compared with the background value of Tianjin soil^42^, the Cd and Pb contents in each sample exceeded the corresponding background values by 2.03–5.39 and 1.18–1.77 fold, respectively. The result of the two-tailed t-test in SPSS showed that the contents of Cd and Pb in all samples were significantly higher than the corresponding background values (p < 0.01). In addition, the contents of Zn, Cr, Cu, Mn, Ni, and V in several samples were higher than the corresponding background values, which accounted for 37.5%, 18.8%, 18.8%, 10.4, 4.2%, and 4.2%, respectively. The coefficient of variation (CV) characterizes the spatial variability of heavy metals in soil^45^. The CV values of Cd, Cu, Ni, and Zn ranged from 20 to 50%, which corresponded to the category of moderate variation and indicated the existence of localized sources that caused the spatial diversity of these heavy metals.

**Table 2:** Statistical description of heavy metals in soils of the research area.

	Min mg/kg	Max mg/kg	Mean ± SD mg/kg	CV%	TJBV mg/kg	SL mg/kg
Cd	0.18	0.48	0.26 ± 0.06	23.84	0.09	0.3
Cr	63.52	91.45	78.99 ± 6.63	8.39	84.2	200
Cu	14.65	64.99	25.14 ± 9.46	37.61	28.8	100
Ni	14.07	50.26	21.53 ± 5.76	26.75	33.3	100
Pb	24.83	37.15	29.92 ± 2.90	9.68	21.0	120
Zn	52.76	197.17	79.57 ± 24.98	31.39	79.3	250
V	58.45	98.07	70.48 ± 8.87	12.58	85.2	–
Mn	475.90	842.58	596.90 ± 80.03	13.41	686	–

–: no data available, *SD* standard deviation, *CV%* coefficient of variation, *TJBV* soil background values of Tianjin, *SL* screening values in CNS (GB 15618-2018) (6.5 < PH ≤ 7.5).

**Figure 2:**
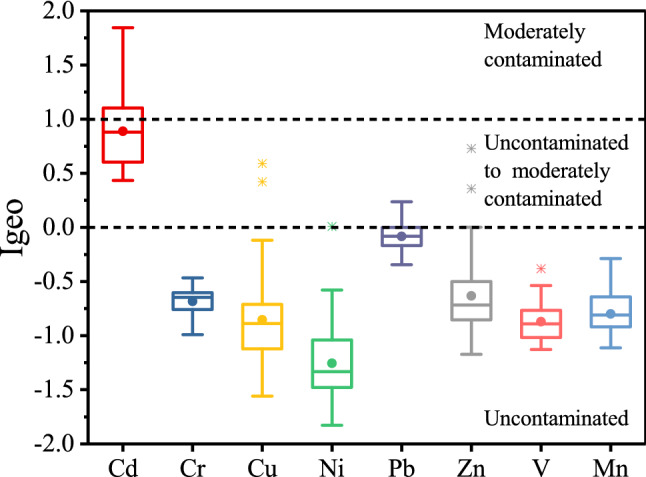
Igeo values for heavy metals in the research area.

As shown in Fig. 2, within the research area, Cd Igeo values at different sampling sites ranged from 0.44 to 1.85; 28 sites belonged to the category “uncontaminated to moderately contaminated”, which accounted for 58.33% of the sampling sites (see supplementary Table S1). The other 20 sampled locations belonged to the category of “moderately contaminated”, which accounted for 41.67% of the sampled locations. Pb Igeo values ranged from − 0.34 to 0.24, with 12 sites belonging to the category of “uncontaminated to moderately contaminated”, which accounted for 25.00% of the sampled locations. Most of the Igeo values for Zn, Cu and, Ni were negative, with 3, 2 and 1 sampled locations belonging to the category of “uncontaminated to moderately contaminated”, respectively. The rest of the heavy metals had negative Igeo values in all sampled locations. In general, the number of positive Igeo values for Cd and Pb were higher than those for other heavy metals, and the accumulation degrees of these two heavy metals relative to the corresponding soil background levels were more significant than those of other heavy metals, indicating the influence of exogenous inputs on Cd and Pb.

By combining the results of descriptive statistics (i.e., CV and Igeo of heavy metals), Cd was shown to exhibit the highest accumulation level in the agricultural soil, and this was affected by localized sources. The accumulation level of Pb was slightly lower than that of Cd and exhibited a relatively even distribution feature. Other heavy metals had lower accumulation levels, indicating mild pollution within the research area. Nevertheless, in the determination results of vegetable samples we collected simultaneously in the research area, we found that the Pb, Cr, and Cd contents of numerous vegetable samples exceeded the corresponding threshold determined in the Chinese National Food Safety Standard (GB 2762-2017). Our results indicated that vegetables in the research area were contaminated by heavy metals, and it is necessary to analyze the sources of heavy metals in soil.

### Correlation analysis results

To ensure the rationality of the source analysis, other simultaneously determined elements, including Ca, Fe, Mg, P, and Ti, were applied in data analyses. The correlation coefficient matrix is shown in Table 3. The correlation coefficients between Ca and Mg, Ca and Ni, Mg and Ni were 0.64, 0.73, and 0.75, respectively, indicating high statistical significance. Ca and Mg are typical crustal elements with high background values in the soil of the research area, indicating that their possible source was soil parent material. P is one of the essential nutrients for plant growth and is mainly derived from the application of phosphate fertilizer. The results showed that P was significantly correlated with Cu and Zn, with correlation coefficients of 0.66 and 0.76, respectively. Cu and Zn were closely correlated with a correlation coefficient of 0.91. Additionally, Cd was correlated with Zn and Cu with correlation coefficients of 0.46 and 0.44, respectively. Our results were consistent with the conclusions of previous studies, which demonstrated that Cu and Zn were related to phosphate fertilizer^46,47^, and Cd was closely related to the nonferrous metal metallurgy industry^34^. The correlation coefficient between Fe and Mn levels was 0.95, indicating high statistical significance. Our field investigation showed that the irrigation sewage of Wuqing District contained significant amounts of Fe and Mn, indicating that sewage irrigation was a potential source of these two heavy metals.

**Table 3:** Correlation coefficients matrix of elements (N = 48).

	Ca	Cd	Cu	Fe	Mg	Mn	Ni	P	Pb	Ti	V	Zn
Ca	1.00											
Cd	0.09	1.00										
Cu	0.16	0.44**	1.00									
Fe	0.31*	−0.15	0.26	1.00								
Mg	0.64**	0.03	0.29*	0.85**	1.00							
Mn	0.32*	0.01	0.44**	0.95**	0.83**	1.00						
Ni	0.72**	−0.10	0.18	0.64**	0.75**	0.62**	1.00					
P	−0.05	0.63**	0.66**	−0.15	−0.04	0.09	−0.17	1.00				
Pb	0.05	0.08	0.31*	0.76**	0.52**	0.78**	0.37**	0.14	1.00			
Ti	0.03	−0.25	−0.04	0.74**	0.52**	0.67**	0.33*	−0.31*	0.55**	1.00		
V	0.27	−0.18	0.21	0.97**	0.77**	0.91**	0.64**	−0.20	0.77**	0.82**	1.00	
Zn	0.12	0.46**	0.91**	0.12	0.16	0.32*	0.06	0.80**	0.26	−0.15	0.07	1.00

Levels of significance: *p < 0.05. **p < 0.01.

### PCA results

**Figure 3:**
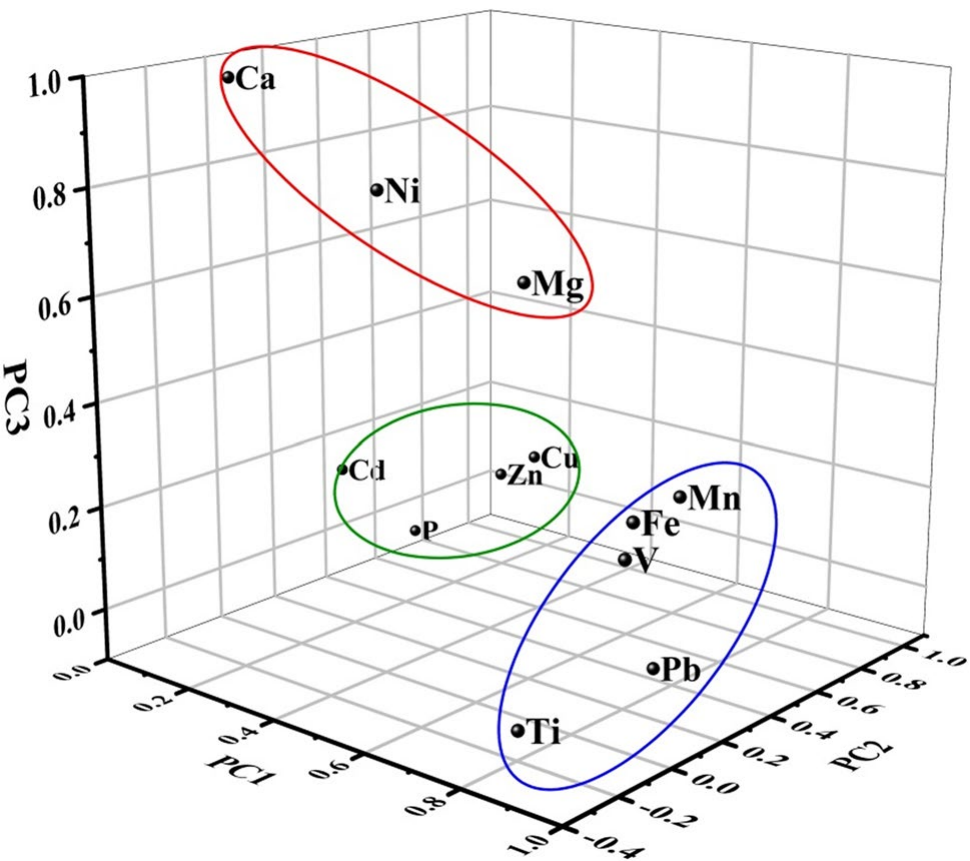
Three-dimensional scatter point load diagram based on all PCs.

The dataset we used in this study passed the Kaiser–Meyer–Olkin (KMO) and Bartlett sphericity tests (KMO = 0.73 > 0.6, P = 0.00 < 0.01), indicating the suitability of the data for PCA. As shown in Table 4 and Fig. 3, three PCs were produced with an initial eigenvalue above 1, which represented 85.11% of the total variance. PC1 (explaining 39.15% of the total variance) mainly comprised Mn (0.90), Fe (0.93), Pb (0.85) and V (0.94). Irrigation sewage used in the research area contained high amounts of Fe and Mn and would constitute an external source for the agricultural soil. Pb is a major tracer of traffic emissions due to the combustion of gas and the wear of engines^48,49^. Massive roads are evenly distributed within the research area, causing Pb, which is mainly discharged by vehicles, to deposit uniformly on farmland. This inference was supported by the lower CV of Pb. Therefore, PC1 was dominated by both sewage irrigation and transportation. Cu (0.87), P (0.92), Zn (0.93), and Cd (0.71) were strongly associated with PC2 (explaining 26.24% of the total variance). As the major element of phosphate fertilizer, a massive amount of P could be imported into agricultural soil due to its wide application in this area. Studies have shown that industrial emissions and fertilizer application can both enhance Cu, Zn and Cd levels in the agricultural soil of Wuqing district^39^. Thus, PC2 can be attributed to a combination of agricultural practice and industrial emissions. Moreover, PC3 (explaining 19.72% of the total variance) had the highest loadings of Ca (0.96), Mg (0.68), and Ni (0.82). Crustal elements Ca and Mg were mainly affected by the weathering processes of soil and rock, whereas Ni could be attributed to parent material due to its low accumulation relative to background level. Therefore, PC3 represents soil parent material.

**Table 4:** Loadings of elements on VARIMAX-rotated factors of different datasets.

	PC1	PC2	PC3
Ca	0.01	0.06	0.96
Cd	−0.16	0.71	0.04
Cu	0.24	0.87	0.13
Fe	0.93	−0.02	0.33
Mg	0.65	0.09	0.68
Mn	0.90	0.219	0.32
Ni	0.42	−0.05	0.82
P	−0.09	0.92	−0.13
Pb	0.85	0.22	−0.01
Ti	0.85	−0.28	−0.01
V	0.94	−0.08	0.28
Zn	0.13	0.93	0.05
Proportion of variance%	39.15	26.24	19.72
Cumulative proportion%	39.15	65.39	85.11

### PMF results

During the utilization of PMF, the optimal result is generally determined by finding the minimum Q, residuals normally distribute between − 3 and 3, and higher correlation coefficients are produced when comparing observed and predicted values (r^2^)^36,50^. Appropriate selection of the number of factors and identification of the characteristic sources of the elements depend only on field investigation information and other literature. Using this method individually can generate a subjective result. To ensure the reliability of the PMF results, we combined the results of correlation analysis and PCA to provide bases for PMF analysis. Considering that the number of PCs was 3, the number of PMF factors was initially set to 3, 4, and 5, the start seed number was randomly obtained, the number of runs was 20, and each run converged (see supplementary Table S2). The minimum and most stable Q value was found when the factor number was set to 4, with five residuals distributed out of the range −3 to 3, and the prediction r^2^ ranged from 0.68 to 0.98, indicating that the four-factor PMF solution was rational.

**Figure 4:**
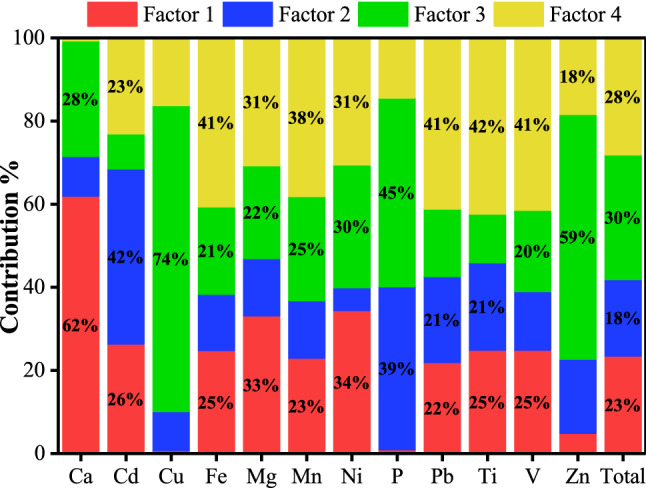
Source contributions analyzed by PMF.

The results of PMF analysis are shown in Fig. 4. Factor 1 showed the most significant relative contributions to Ca, Mg, and Ni levels (61.91%, 33.11%, and 34.37%, respectively). Ca and Mg are typical crustal elements that have high background values in the research area and are highly correlated with each other, indicating that they can represent the soil background. In addition, only Ca, Mg, and Ni were observed with high loadings in PC3 (0.96, 0.68, and 0.82, respectively), which represented soil parent material. Taking these results into account comprehensively, Factor 1 can be classified as a soil parent material source.

Factor 2 was characterized by Cd (42.14%). Studies have shown that the major sources of Cd are industrial emissions and agricultural practices^50,51^. Since P is the main element of fertilizer, heavy metals that were closely correlated with P could have mainly originated from fertilizer. The coefficients for correlation of P with Zn, Cu, and Cd decreased in the order listed (0.80, 0.66, and 0.63, respectively), which indicated that the influence of fertilizer application on Cd was weaker than those on Cu and Zn. Moreover, the contribution to P level was not dominant in Factor 2, suggesting that Factor 2 is mainly affected by industrial sources.

Factor 3 was dominated by P, Cu, and Zn levels (45.40%, 73.64%, and 58.85%, respectively). Studies have shown that large quantities of Cu and Zn can be introduced to agricultural soil upon the application of phosphate fertilizer^52,53^. Additionally, the utilization of livestock manures and pesticides has also contributed to Cu and Zn levels in agricultural soil^54^. According to the close interaction between these three elements (P–Cu: 0.66, P–Zn: 0.80, Cu–Zn: 0.91) and high loadings in PC2 (P: 0.92, Cu: 0.87, Zn: 0.93), Factor 3 can be as attributed to agricultural practices.

Factor 4 was weighted primarily on Pb, V, Fe, and Mn (41.18%, 41.49%, 40.66%, and 33.61%, respectively). These four heavy metals closely interacted, with correlation coefficients ranging from 0.76 to 0.97, and exhibited high loadings in PC1. Although unleaded gasoline has been used nationwide, gasoline is still the main source of Pb pollution in soil. The wear of an automobile braking system and wheel balancer also lead to Pb discharges from traffic^55^. Fe and Mn were strongly correlated with one crustal element Mg (the coefficients were 0.85 and 0.83, respectively), but weakly correlated with the other crustal element Ca (the coefficients were 0.31 and 0.32, respectively), indicating that they did not mainly originate from soil parent material. Additionally, these two elements were abundant in the irrigation sewage of research area. Thus, Factor 4 is considered a mixed source derived from transportation and sewage irrigation.

The contribution rates of Factor 1, Factor 2, Factor 3 and Factor 4 were 23.46%, 18.39%, 29.97% and 28.17%, respectively. The categorizations of each element analyzed by PCA and PMF were almost identical, and the correlations of elements can explain the source apportionment results for each element analyzed by PMF. The application of correlation analysis and PCA results remedy the shortcomings of the PMF running process, provide numerical bases for determining the categories of factors, and strengthen the accuracy of identifying factor sources.

### Results of the Cd and Pb isotope ratio method

The heterogeneities between the isotopic compositions of different potential pollution sources forms the basis for using the isotope technique in source analysis^56^. One-way ANOVA indicated that the diversities among the ^111^Cd/^114^Cd, ^111^Cd/^112^Cd, ^208^Pb/^206^Pb, and ^207^Pb/^206^Pb ratios of different potential sources were significant (P < 0.05), which illustrated that the isotope ratios of these potential sources could be used to analyze the sources of Pb and Cd.

**Figure 5:**
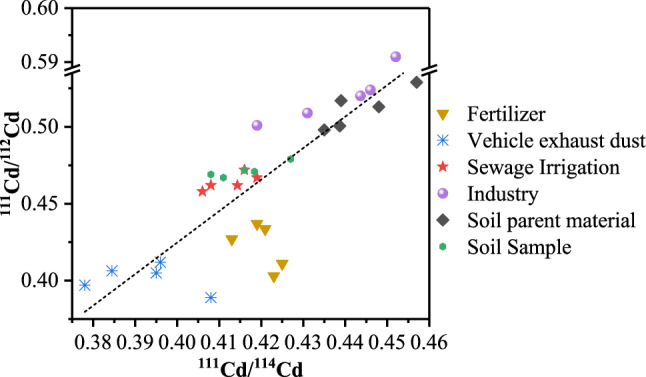
Cd isotope ratios of soil and potential source samples in the research area.

**Figure 6:**
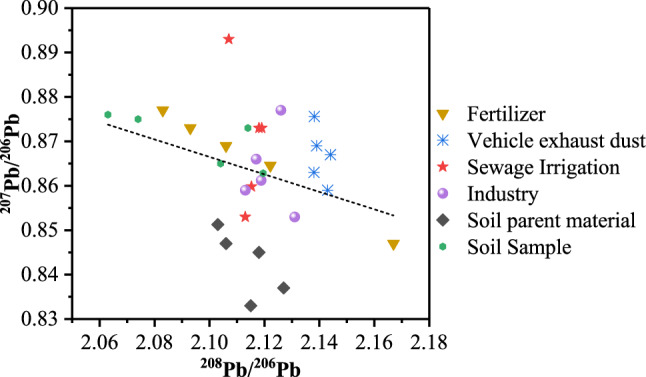
Pb isotope ratios of soil and potential source samples in the research area.

The isotope graphs in Figs. 5 and 6 display the Cd and Pb isotopic compositions of soil and potential source samples in the research area, respectively. The average ratios of ^111^Cd/^114^Cd, ^111^Cd/^112^Cd, ^208^Pb/^206^Pb and ^207^Pb/^206^Pb in all potential sources ranged from 0.3845 to 0.4436, 0.4063 to 0.5202, 2.1030 to 2.1382 and 0.8513 to 0.8756, respectively. These narrow variation ranges for Cd and Pb isotope ratios indicated that the variations among contributions from different sources of Cd and Pb were inconsiderable in the research area. As shown in Fig. 5, the Cd isotope ratios of soil samples adjacently ranged between parent material soil, industry soil, and irrigation sewage water and were scattered from fertilizer and vehicle exhaust dust, indicating that Cd was mainly affected by soil parent material sources, industrial sources, and sewage irrigation sources and were less affected by agricultural sources and transportation sources. As shown in Fig. 6, the Pb isotope ratios of soil samples adjacently ranged between fertilizer, industry soil, and irrigation sewage water and showed scatter from parent material soil and vehicle exhaust dust, indicating that Pb was mainly affected by agricultural sources, industrial sources, and sewage irrigation sources and less affected by soil parent material sources and transportation sources.

Table 5 shows the contribution rates of each source calculated by EPA-IsoSource based on the average Cd and Pb isotope ratios of agricultural soil and potential source samples. For Cd, agricultural sources and transportation sources had relatively lower contribution rates of 16.40% and 15.20%, respectively. The diversities among the contribution rates for sewage irrigation sources, industrial sources, and parent material sources were not obvious, with contribution rates of 24.70%, 20.90%, and 22.70%, respectively. The summed contribution rate of these three sources was 68.30%, indicating their relatively substantial effect on Cd accumulation in the research area. For Pb, parent material sources and transportation sources had relatively lower contribution rates of 19.40% and 19.00%, respectively, compared to 20.50–20.60% for sewage irrigation sources, industrial sources, and agricultural sources. The summed contribution rate of these three sources was 61.60%, indicating their relatively considerable effect on Pb accumulation in the research area. However, the difference between the contribution rates of potential sources was below 10%, indicating that no single source dominated Cd and Pb contamination. The differences among the contribution rates of sources analyzed by the Cd and Pb isotope ratio method were less than 5%, indicating the uniformity of the source contributions determined by Cd and Pb isotope ratios.

**Table 5:** Contribution rates of different sources base on average isotope ratios of Cd and Pb.

Pollution source	^111^Cd/^114^Cd	^111^Cd/^112^Cd	Contribution rate (%)	^208^Pb/^206^Pb	^207^Pb/^206^Pb	Contribution rate (%)
Agricultural sources	0.42093	0.43358	16.40	2.12215	0.86455	20.50
Transportation sources	0.38447	0.40633	15.20	2.13817	0.87561	19.00
Sewage irrigation sources	0.41434	0.46191	24.70	2.11527	0.85983	20.50
Industrial sources	0.44361	0.52019	20.90	2.11877	0.86121	20.60
Parent material sources	0.43868	0.50057	22.70	2.10299	0.85127	19.40

### PMF verification based on lead isotopes

**Figure 7:**
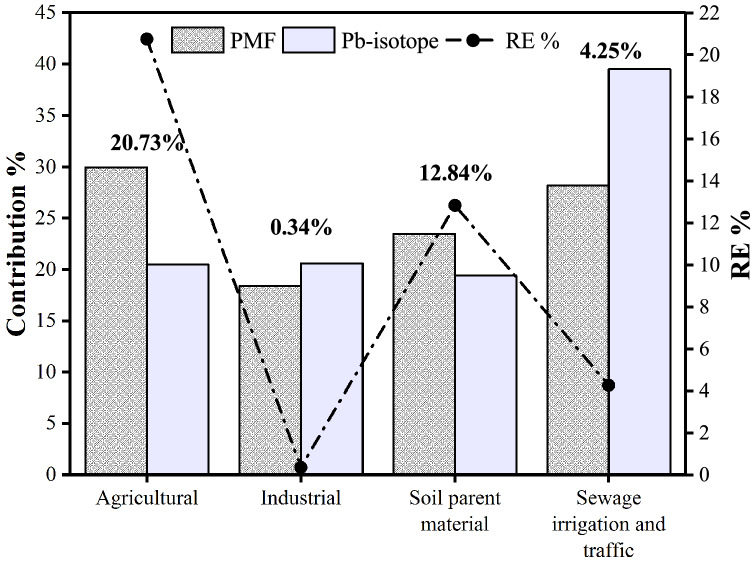
REs between source contribution rates analyzed by PMF and Pb isotope ratio methods.

Correlation analysis and PCA were applied to verify the running results of PMF and improve the accuracy of source category identification. However, since correlation analysis and PCA cannot analyze the contribution of each source accurately, it is necessary to verify the contribution rates determined by PMF. Considering the comparatively high resistance to fractionation of Pb isotopes, it has been widely used to analyze the sources of Pb pollution in environmental media^57,58^. Therefore, the Pb isotope method was selected to verify the rationality of the source contribution rates determined by PMF. As shown in Fig. 7, the source contribution rates for Pb determined by the PMF and isotope ratio methods were close to each other. The REs of the contribution rates of sources to Pb calculated by the PMF and isotope methods were 20.73% for agricultural sources, 0.34% for industrial sources, 12.84% for parent material sources, and 4.25% for mixed sources of sewage irrigation and transportation. REs < 40% indicated that the source contribution result from the PMF model was reliable^59^. The REs of agricultural sources and soil parent material sources were higher than those of industrial sources and mixed sources from transportation and sewage irrigation, which indicated that PMF could generate bias in analyzing the contribution of various pollution sources.

## Conclusion

In this study, the sources of heavy metal pollution in agricultural soils in the sewage irrigation area of Wuqing, Tianjin were analyzed by applying multiple methods. Heavy metal pollution of agricultural soil in the research area was mild except for that of Cd and Pb. The aggregate result of correlation analysis, PCA, and PMF indicated that soil parent material sources (23.46%) contributed significantly to Ca, Mg, and Ni pollution, industrial emission sources (18.39%) contributed significantly to Cd pollution, agricultural practice sources (29.97%) contributed significantly to P, Cu, and Zn pollution, and mixed sources of sewage irrigation and transportation (28.17%) contributed significantly to Fe, Mn, Pb, and V pollution. The contribution rates of each source quantified by the Cd and Pb isotope ratio method were similar, indicating that no single source dominated the Pb and Cd pollution. The REs between the contribution rates of Pb sources analyzed by PMF and Pb isotopes were lower than 40%, suggesting the rationality of the PMF contribution results. Taken together, the results suggest that PMF can be effectively applied to source analysis of heavy metal pollution in agricultural soil, whereas correlation analysis and PCA should be utilized to reduce the fuzziness in obtaining reasonable PMF results and produce bases for defining the source types of each factor. After obtaining the contributions from various sources, the isotope ratio method should be applied to verify the accuracy of source contribution rates determined by PMF.

## Supplementary Information

Supplementary Tables.

## Data Availability

The datasets analyzed during the current study are available from the corresponding author on reasonable request.
