# Supplementary material for: Source analysis of heavy metal pollution in agricultural soil irrigated with sewage in Wuqing, Tianjin
Source: Sci Rep. 2021 Sep 8;11:17816. doi: 10.1038/s41598-021-96367-8 (PMC8426478; doi:10.1038/s41598-021-96367-8)
Supplement: Supplementary file 1 — Supplementary Tables. [file 41598_2021_96367_MOESM1_ESM.pdf]

Source analysis of heavy metal pollution in agricultural soil irrigated with sewage in Wuqing, Tianjin

Supplementary Information

Jingran Wang[1], Danyang Yu[1,5], Yanhong Wang[1,2,3,*], Xueli Du[1], Guochen Li[1,3], Bo Li[1,3], Yujie Zhao[4], Yinghui Wei[1] & Shuang Xu[1]

[1]Institute of Applied Ecology, Chinese Academy of Sciences, Shenyang, 110016, China
[2]Key Laboratory of Pollution Ecology and Environmental Engineering, Institute of Applied Ecology, Chinese Academy of Sciences, Shenyang, 110016, China
[3] Liaoning Engineering Technology Research Center of Agricultural Products Quality and Environment Safety Control, Institute of Applied Ecology, Chinese Academy of Sciences, Shenyang, 110016, China
[4]Key Laboratory for environmental factors control of Agro-product quality safety, Ministry of Agriculture and Rural Affairs, Tianjin, 300191, China
[5] Shenyang University of Chemical Technology, Shenyang, 110142, China
*Correspondence: wangyh@iae.ac.cn

Table S1. Statistical of Igeo classification (n=48)

		Cd	Cr	Cu	Ni	Pb	Zn	V	Mn
Unpolluted(Igeo≤0)	amount	0	48	46	47	36	45	48	48
	proportion(%)	0	100	95.83	97.92	75.00	93.75	100	100
From unpolluted to moderately polluted(0<Igeo≤1)	amount	28	0	2	1	12	3	0	0
	proportion(%)	58.33	0	4.17	2.08	25.00	6.25	0	0
Moderately polluted(1< Igeo≤2)	amount	20	0	0	0	0	0	0	0
	proportion(%)	41.67	0	0	0	0	0	0	0

Table S2. Q(Robust) and Q(True) value of converged 20 Runs

Run #	Q(Robust)	Q(True)	Converged
1	429.3	446.824	Yes
2	429.293	446.83	Yes
3	429.292	446.832	Yes
4	429.295	446.826	Yes
5	429.293	446.829	Yes
6	429.27	446.842	Yes
7	429.278	446.839	Yes
8	429.297	446.823	Yes
9	429.288	446.832	Yes
10	429.253	446.851	Yes
11	429.291	446.83	Yes
12	429.275	446.843	Yes
13	429.282	446.837	Yes
14	429.292	446.829	Yes
15	429.267	446.847	Yes
16	429.28	446.834	Yes
17	429.316	446.817	Yes
18	429.287	446.831	Yes
19	429.288	446.83	Yes
20	429.296	446.825	Yes
